# Assessing and Improving Study Skills Support in Medical Education Through a Student-Staff Partnership: Mixed Methods Approach

**DOI:** 10.2196/65053

**Published:** 2025-09-03

**Authors:** Nicole Tay, Anaïs Deere, Dhivya Ilangovan, Carys F E Phillips, Emma Kelley

**Affiliations:** 1 UCL Medical School, University College London, 74 Huntley Street, London, WC1E 6DE, United Kingdom, 44 02031088235

**Keywords:** study skills, medical education, E-learning, student-staff partnership, student perspectives, students, studying, mixed methods, questionnaire, thematic analysis, school, learning, exams, medical school, teaching, education

## Abstract

**Background:**

The necessity for self-regulated, lifelong learners in the rapidly evolving field of medicine underscores the importance of effective study skills. Efforts to support students with these skills have had positive outcomes but are often limited in scope and accessibility, with a tendency to target groups facing immediate challenges.

**Objective:**

This study aimed to explore the student perspective on study skills support at University College London Medical School through a student-staff partnership, with the goal of guiding future improvements.

**Methods:**

A mixed methods approach was adopted using an anonymous questionnaire and focus groups. After analyzing questionnaire responses using descriptive statistics to refine focus group questions, focus groups were conducted to delve deeper into identified issues. Transcripts were analyzed thematically using inductive coding.

**Results:**

In total, 116 students completed the questionnaire in full and 6 students participated in 2 focus groups. The questionnaire revealed that 68% (68/100) of respondents felt that they never received study skills support at University College London Medical School. Preferred methods of support included small group sessions (56/100, 56%) and topics like examination preparation (83/100, 83%) and study skills specific to medicine (72/100, 72%). Focus group themes were the lack of current study skills support, delivery of study skills support, specific study skills for medical school, personalized approach to support needed, and accessing support. Findings informed the co-creation of study skills resources.

**Conclusions:**

Overall, the findings highlight the need for strategically incorporating study skills support at medical school, emphasizing early and consistent promotion and tailored delivery methods.

## Introduction

In the dynamic realm of modern medicine, the need for practitioners to be self-regulated, lifelong learners is imperative if they are to keep up with ever-evolving medical knowledge [1]. In White et al’s evidence-based model [2], self-regulated learning (SRL) is composed of a set of learnable skills: “planning, learning, assessment, and adjustment,” acquisition of which can be facilitated by educators. Study skills, or “learning strategies,” have been identified as an important component of the learning phase of SRL, and they can be defined as an “integrated repertoire of tactics and strategies, which facilitate acquisition, organization, retention, and application of such knowledge” [3].

SRL and study skills are relevant across higher education, and their importance is especially pronounced in medical education which incorporates theoretical and practical knowledge to develop graduates with the ability to learn independently and adaptively throughout their future careers [4,5]. In the clinical environment, SRL has been positively associated with students’ academic achievement, clinical skills performance, and mental health outcomes [6]. SRL strategies have also shown a particularly strong association with affective outcomes such as attitude, motivation, and confidence [7], suggesting that SRL may support not only learning performance but also how students feel about their learning. The association between SRL strategies and learning outcomes is more pronounced in clinical clerkship than in preclerkship phases [7], and literature suggests that support for SRL should be more explicit and structured earlier in training [8,9]. This highlights the importance of preparing students to self-regulate before they enter the clinical environment. This may be best supported by approaching SRL as a shared endeavor from the outset, with medical schools playing an active role in its early development [8,10].

The broader literature highlights the value of educational interventions to support SRL in medical students, such as tailored workshops [11] and mentor guidance [10]. Classroom-based SRL interventions and one-on-one academic coaching have also been positively received by first-year students and linked to greater anticipated use of SRL strategies [12]. In clinical settings, factors such as access to full-time clinical teachers, peer collaboration, and mentor support have been identified as significant predictors of SRL and are associated with improved clinical performance [13]. However, study skills interventions in medical education often follow a reactive-deficit model that targets students who have already encountered difficulties, such as those at risk of academic failure [14] or academically low-achieving [15], or a proactive-deficit model that focuses on students deemed “at risk,” such as new entrants to medical school [16]. While identifying and supporting students in need is essential, this approach may inadvertently exclude students across the wider academic spectrum. Evidence indicates that study strategies influence academic achievement across the performance spectrum [17-19], supporting the case for more universal and inclusive approaches to study skill development.

At University College London Medical School (UCLMS), the MBBS program is a 6-year integrated degree, including an intercalated BSc in the third year [20]. The curriculum comprises themed modules, with clinical and professional practice running vertically throughout the program. Year 1 and 2 modules focus on the fundamentals of clinical science, and Years 4 to 6 modules emphasize clinical practice across specialties [21]. At the time of the study, UCLMS offered an array of study skills resources which supported SRL, such as “study skills clinics” (one-to-one or small group sessions with clinical staff members), support from personal tutors, and online learning resources. University College London (UCL) also provided study skill support accessible to all students such as online academic resources [22], alongside broader institutional support for students with specific learning differences or long-term health conditions, including tailored accommodations for assessments and teaching settings [23].

Despite the range of available support, a 2020 internal survey at UCLMS revealed that many students felt undersupported in developing effective study skills. This perceived gap between resource availability and student experience signaled a need to evaluate and improve current provision. In response to this, a student-staff partnership (the authors) was formed to explore and address the perceived gap in study skills support. Although student input into medical curriculum design is often limited or restricted to a needs analysis [24], literature suggests that engaging learners in the design process through student-staff partnership can enhance learning engagement, teaching effectiveness, and assessment outcomes [25]. This project adopted a student–staff partnership model to facilitate collaborative educational research that would not only develop the research skills of the team but also support a more authentic, peer-informed exploration of the student voice. By positioning students as coresearchers, supported by faculty mentorship and institutional resources, the partnership aimed to generate deeper insights into the student experience [26].

As interest in active student involvement in medical education grows [27], establishing models for successful collaboration is increasingly important. As a result, this research project aimed to use the student-staff partnership to explore the student perspective on study skills support at medical school, using findings to cocreate relevant resources and guide future improvements.

## Methods

### Overview

**Figure 1.**
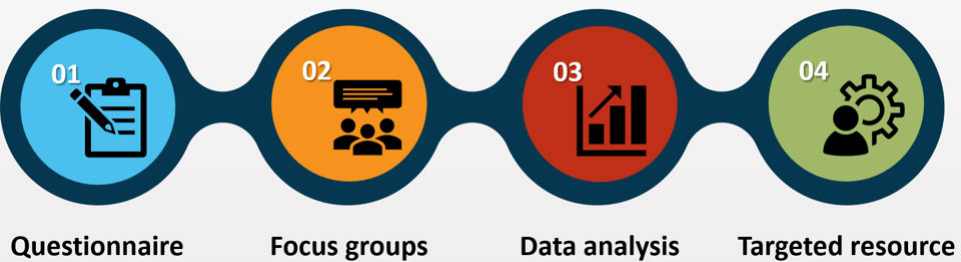
Summary of the methods.

This is a mixed methods study consisting of a questionnaire and focus group, as depicted in Figure 1. A mixed methods design enabled collection of both qualitative and quantitative data, which allowed for greater confidence in the validity of any findings by means of triangulation [28], which was felt to be important given the relative lack of research in this specific area. This factor is also what led to the decision of conducting focus groups, due to their usefulness in exploratory research [29].

An interpretivist paradigm was adopted for this study, recognizing that reality is subjective and socially constructed  [30]. Aligned with social constructivism, this approach emphasizes the importance of understanding participants’ experiences within their educational environment. It informed the use of focus groups and inductive thematic analysis to explore how students construct meaning based on their individual experiences. The collaborative student–staff research model further reflects the interpretivist emphasis on coconstructed knowledge, positioning students as active contributors to the research process [26].

### Questionnaire

#### Sampling Procedure and Recruiting Study Participants

The methodology of the web-based questionnaire is described in line with the CHERRIES checklist [31]. A web-based questionnaire was designed and distributed electronically, by purposive sampling of UCLMS medical students. Due to the diversity of intercalated BSc courses completed by students in their third year and the varying study skill requirements they entail, only students in Years 1, 2, 4, 5, and 6 of the MBBS program were recruited for this study.

#### Quality Assurance

A preliminary version of the questionnaire was developed by 2 students in the partnership and subsequently reviewed by the rest of the team. It was then further evaluated by the UCL Changemakers team, revised accordingly, and piloted by the other 2 students and 2 staff members within the partnership.

#### Recruitment Process

Students were initially sent a link to the questionnaire by email from the medical school, with a reminder email sent 1 month later. To further promote participation and increase response rates, online platforms such as WhatsApp [32] were also used. These channels were chosen due to their popularity among students and the observation by the medical student authors that their peers were particularly responsive through these platforms [33].

#### Survey Administration

The questionnaire was held on Opinio, accessible via link in the invitation email. As the study was voluntary, financial incentives were used to try and increase engagement [34]; students could enter a draw to win vouchers, which were funded by UCL Changemakers. The survey was anonymized with a separate link provided to enter the prize draw upon completion of the questionnaire. Data collection took place from March to May 2022.

#### Study Design

The questionnaire was structured to allow students to reflect on their current study skills, gather information on which existing UCL resources they accessed and their perceived effectiveness, and explore students’ preferences for the delivery of future study skills support. It consisted of a total of 12 questions with various styles (multiple choice, multiselect multiple choice, Likert scale, and free text). The majority of questions used a 5-point Likert scale to enable participants’ opinions to be quantitatively analyzed.

To facilitate targeted questioning on specific study strategies, seven study skill domains related to self-regulated learning were identified. This framework was developed through a scoping review of relevant literature in study skills and medical education and further refined through collaborative discussions within the research team, as shown in Textbox 1:

Textbox 1. **Summary of the 7 study skill domains related to self-regulated learning.**

- Time management
- Organization
- Exam preparation and technique
- Obtaining reliable sources of information
- Retaining information
- Study skills specific to a medical degree (eg, practical exams and navigating clinical years)
- Study skills and mental health (work or life balance and feeling overwhelmed)

A free space text box was provided near the end of the questionnaire for additional comments or suggestions. The full questionnaire can be found in “Multimedia Appendix 1: Questionnaire”.

It was not mandatory for participants to complete all questions, and they could review and amend their answers before submission. We did not count view rates or participation rates, nor implement techniques which would prevent multiple entries. Partially completed questionnaires were analyzed.

### Focus Groups

Before conducting the focus groups, the research team undertook a collaborative reflexive exercise to acknowledge potential biases during the research process and explore the diverse perspectives within the team [35]. This involved a semistructured discussion led by one of the staff researchers exploring individual assumptions and expectations, recognizing how the medical student researchers’ unique position meant they had their own perceptions of study skills support and desirable outcomes of the project. Also discussed was how this position allowed them to better understand the student body and contextualize their opinions. The staff members also engaged in a separate reflexive discussion to consider their own positionality as educators and contributors to the support systems being evaluated.

Participants were recruited for the focus groups through the questionnaire, with interested individuals emailing the research team directly to maintain questionnaire anonymity. To encourage participation, each focus group attendee received a voucher funded by UCL Changemakers as a financial incentive.

The questionnaire results guided the development of the focus group questions. The focus groups were semistructured to allow students to freely elaborate on their views of study skills support while maintaining enough structure to explore key questionnaire findings in greater depth. To ensure consistency, the interviewers followed an interview schedule with predetermined questions.

Two online focus group interviews were conducted in May 2022 using Zoom [36], a platform widely used for synchronous lectures and small-group teaching at UCLMS, to help participants feel comfortable engaging and turning on their cameras. Both focus groups were facilitated by 2 medical students from the research team to foster open, honest discussion and encourage participants to freely critique this study skills support without staff members present [37].

The focus groups began by inviting students to share their understanding of the term “study skills,” with clarification provided as needed to ensure a clear understanding of the discussion topic. Respondents were asked a series of open-ended questions to establish their experience of study skills support at UCLMS, share their views on preferred methods of study skills support delivery, and explore further results of the questionnaire. Although questionnaire results were not shared with participants beforehand, they were introduced and explored during the discussions. The semi-structured focus group questions can be found in “Multimedia Appendix 2: Focus Group Questions”.

Focus groups were video recorded and subsequently deleted after verbatim transcription by 2 of the authors, with all identifying information removed in accordance with the consent form the participants had signed before.

### Analysis

Quantitative data from the survey were analyzed using descriptive statistics to compute percentages, means, and frequencies. Qualitative data from the focus groups were analyzed using inductive coding guided by Braun and Clark’s phases of thematic analysis [38]. This process was informed by social constructivist principles, acknowledging that themes and insights emerge through the interactive process of data collection and analysis. Following familiarization with the transcripts through repeated readings, the questions were evenly distributed among the medical student researchers and coded independently. Initial codes were then reviewed collaboratively in meetings to standardize their interpretation across all questions. Each student researcher also reviewed and analyzed the coding completed by the other researchers to further ensure consistency. Through subsequent discussions, the codes were grouped into broad themes based on similarity, with subthemes developed where further categorization was needed. The codes and themes were then reviewed by the entire research team to refine them into more clearly defined categories. This process was supported by the program NVivo.

### Ethical Considerations

Ethics approval for the study was granted by the Changemakers team—an initiative that provides funding and support for collaborative projects between students and staff aimed at enhancing the student learning experience at UCL (ethics approval number 12385/001). Informed consent was obtained from all questionnaire participants. No personally identifiable information was collected, aside from participants’ year of study. All data were securely stored on a password-protected platform, accessible only to the research team. All data collected was anonymized in both the questionnaire and the focus group interviews. Students participating in the online questionnaire were given an option to partake in a USD $26.80 prize draw after completion; which was awarded to 1 student. Students participating in the focus group interviews all received compensation of USD $13.40.

## Results

### Questionnaire Results

In total, 1682 students across 5 years of medical school at UCL were sent the questionnaire. In total, 116 students out of 1682 (6.9% response rate) completed the online questionnaire in full; there was a 13.8% (16/116) drop-out rate from the start to the end of the survey. There was a UCL Central Study Skills Page available to all students for support, and 80% (80/100) of respondents indicated they had not accessed it. Among the 20% (20/100) of respondents who had accessed the page, its effectiveness was rated with a median score of 3/5.

A total of 68% (68/100) of students reported they never received study skills support during their time at UCLMS. Among the 32% (32/100) who reported they had received study skills support, the majority (81%, 26/32) indicated they received support 1 to 2 times a year.

When participants were asked to evaluate the effectiveness of various study skills delivery methods, all approaches—including lectures, self-directed learning, one-to-one support from staff or peers, personal tutors, and transition mentors (who provide weekly teaching sessions to support students’ adjustment to university education in Years 1 and 2)—received a median effectiveness rating of 3 out of 5 across all year groups. In contrast, the study skills clinic received a lower median rating of 2.

The majority (56%, 56/100) of respondents indicated a preference for small group study skill sessions when asked about their preferred methods of delivery. Lectures (43%, 43/100), one-to-one support from staff (41%, 41/100), one-to-one support from peers (18%, 18/100), peer-to-peer support (34%, 34/100) and the study skills clinics (33%, 33/100) were also popular options. The 4 most popular topics preferred by students to be included within study skills support were: exam preparation (83%, 83/100), study skills specific to a medical degree (72%, 72/100), retaining information (69%, 69/100), and taking in new information (58%, 58/100). The demographics and summary of the questionnaire results can be found in the “Multimedia Appendix 3: Questionnaire results”.

### Focus Group Results

A total of 6 students participated in 2 focus groups: separated by those in Years 1 to 2 of study and those in Years 4 to 6 of study. This reflects that changes in SRL occur in a clinical learning environment [6], so the approach to study skills may differ in these groups as the modules in Years 4 to 6 are more focused on clinical practice.

Three individuals participated from Years 1 and 2, and three individuals participated from Years 4 to 6. Each focus group interview lasted around 40 minutes. Analysis identified 5 themes that explored the student perspective on study skills support: lack of current study skills support, delivery of study skills support, specific study skills for medical school, personalized approach to support needed, and accessing support.

#### Theme 1: Lack of Current Study Skills Support

##### Subtheme 1.1: - Lack of Support

In general, students felt there was a lack of study skills support provided by the University. This was more prominent in the Year 4 to 6 group, where students struggled to give any examples of formal teaching and acknowledged that the majority of their study support came from fellow students.

*... I don’t recall any, at least things I remember, any effective study skills teaching…*

(Participant 2, Year 4 to 6 group)

*... I feel like we haven’t really had that much guidance, it’s mainly from other students.*

(Participant 3, Year 4 to 6 group)

In the Year 1 to 2 group, the problem identified was less to do with the availability of study skills support from the University, as students could provide several examples. Instead, their concern related to the effectiveness of that support.

*... I don’t think it [lecture on learning at medical school] was necessarily something that was particularly well supported or particularly well taught…*

(Participant 1, Year 1 to 2 group)

*... I signed up for it [study skills clinic], but I didn’t end up going because it seemed like it was too generic, like it didn’t seem medicine specific…*

(Participant 2, Year 1 to 2 group)

### Theme 2: Delivery of Study Skills Support

The way in which study skills support should be delivered was discussed. Although no unanimous consensus on the best method of delivery emerged, the advantages and disadvantages of various methods were acknowledged.

#### Subtheme 2.1 - One-to-One Staff Support

One-to-one staff support was a popular method of delivery among the students due to its focus on students’ individual needs. Access to official guidance from medical school personnel, particularly those familiar with the curriculum and examination format, was considered especially beneficial. However, students emphasized the importance of staff fostering an open environment for students to voice their concerns without fear of judgment.

*I think, as long as [the] staff member was kind of open [and] non judgmental and they make it a comfortable atmosphere for you to kind of open up and talk about your issues, I think that’s what we need.*

(Participant 3, Year 1 to 2 group)

#### Subtheme 2.2 - Peer Support

The concept of seeking study skills guidance from peers was positively received, with a preference for support from senior students who had completed the same style of assessments, allowing them to provide tailored, experience-based advice. In addition, while some students acknowledged the existence of established peer support systems in student societies, concerns were raised about the unfairness of the disparity in access to this support for students without such contacts.

*...I feel like that’s kind of unfair and some students also have like seniors who helped them out and stuff and not everyone has that, especially because of covid you know it’s hard to make those contacts.*

(Participant 1, Year 1 to 2 group)

#### Subtheme 2.3 - Small Group Teaching

Small group teaching was generally supported, as students valued the opportunity for interactive discussions and peer exchange of study habits and learning strategies. However, some expressed concern about feeling intimidated by the perceived workload and study strategies of their peers.

*... sometimes I feel like other people are doing so much more than me or studying in so many like more efficient ways and I’m just like really behind and so yeah, I guess, in some ways it helps but in other ways, it can be quite intimidating as well.*

(Participant 3, Year 1 to 2 group)

#### Subtheme 2.4 - Asynchronous vs Synchronous

Students preferred synchronous study skills lectures over asynchronous ones, as the live format was seen to promote better attendance.

*... if it’s asynchronous I don’t think many people would go out of their way to do it because the workload is so much it wouldn’t be anyone’s priority*

(Participant 3, Year 4 to 6 group)

### Theme 3: Specific Study Skills for Medical School

#### Subtheme 3.1 - Medical School Is Unique

Students felt that studying medicine required a different learning approach than both school and other university degrees, particularly due to the unique nature of its assessment methods.

*UCL medicine and medicine in general is really, really different to the way we’re being assessed.*

(Participant 1, clinical group)

*... you can’t just do it [learn] like A-levels or GCSE*

(Participant 2, Year 1 to 2 group)

#### Subtheme 3.2 - Medical School Has a Lot of Content

Students often raised the fact that they felt overwhelmed with the volume of content they were expected to memorize in the course. They also found it challenging to discern the scope and depth of knowledge required, especially as expectations increased with each academic year.

*... we still need to memorize the whole bunch of stuff to apply it and I struggle with that quite a bit just the sheer amount.*

(Participant 2, Year 4 to 6 group)

*I think it’s really difficult to kind of ascertain what exactly we’re expected to know and the level we’re expected to know.*

(Participant 1, Year 4 to 6 group)

### Theme 4: Personalized Approach to Support Needed

#### Subtheme 4.1 - Study Skills Are Personal

Students recognized that not every approach to studying works for everyone as learning styles are very individual.

*... everyone has like different circumstances and different areas that they struggle with; I struggle with time management.*

(Participant 3, Year 1 to 2 group)

#### Subtheme 4.2 - Blind to Own Study Skill Weakness

Students expressed the challenge of identifying weaknesses in their study methods, noting that this awareness is an essential first step toward improvement.

*“I don’t know where I’m going wrong in order to fix it.”*

(Participant 1, Year 4 to 6 group)

### Theme 5: Accessing Support

#### Subtheme 5.1 - Advertising Support

Students felt that study skills services available were not advertised well enough, suggesting promotion through The Royal Free, University College and Middlesex Medical Students’ Association (RUMS), WhatsApp or Instagram (Meta) [20,28]. They also recommended the use of student testimonies so that students could gain a clearer insight into the benefits of the support.

*If you made it a RUMS thing, because if you send it from the med school … I feel like people were kind of hesitant just because you know…I’m gonna be gone after or something. But if you make it like a RUMS thing it’s like a community vibe…*

(Participant 3, Year 1 to 2 group)

#### Subtheme 5.2 - Establishing Support Early On

Students believed that information about study skills support should be offered early in the year as opposed to just prior to exam season, allowing those struggling more time to access and benefit from the assistance.

*... because they can connect earlier on in the year and kind of work on that, for the rest of the year rather than it being something that you do like a month before the exam and just panicking…*

(Participant 3, Year 1 to 2 group)

## Discussion

### Principal Findings

Overall, the results from both the questionnaire and focus groups highlighted a perceived lack of study skills support. There was no unanimous consensus about students’ preferences on how study skills support should be delivered. Despite the availability of established study skills services outlined in the introduction, 68% of questionnaire respondents (68/100) reported never receiving such support. This sentiment was echoed in the focus groups, where students indicated they primarily relied on peer advice for study skills guidance. This reinforces the importance of more personalized study support and the need for review of the promotion and accessibility of existing services.

Lectures, one-to-one support, peer group teaching, and study skills clinics all demonstrated comparable popularity as delivery methods; however, small group study skills sessions were the only format preferred by a majority of students (56%; 56/100) in the questionnaire. Focus group subthemes—“one-to-one staff support” and “small group teaching”—further illuminated mixed perceptions of small group sessions. While small groups facilitated peer sharing of study skills, some students found them intimidating due to comparisons with others. Conversely, whilst one-to-one support from staff was perceived as personalized to students’ individual needs, especially if by a staff member familiar with the curricula and assessment process, its use was contingent on occurring in a nonjudgmental environment. These findings reflect the literature where both classroom-based learning and one-to-one academic coaching are shown to encourage the use of SRL techniques [12].

The focus group themes and subthemes highlighted that each method of study skills support delivery offers distinct advantages and disadvantages, catering to different student priorities. For example, some students valued direct support from staff for its perceived credibility, while others expressed concern about potential judgment when admitting difficulties. This stigma has been previously identified as a barrier towards medical students seeking help, particularly in the context of non-compulsory activities which offer additional support [39]. Moreover, regardless of delivery preferences, students acknowledged that study skills are highly individual, with no “one size fits all” solution—an insight consistent with existing literature [40]. This also aligns with the literature demonstrating the need for individualized support in the clinical environment to accommodate students’ unique SRL strategies [10]. From a faculty perspective, these diverse needs and preferences must therefore be carefully considered when designing study skills support to maximize their effectiveness.

It is clear that social influences shape how students seek study skills support, with many highlighting that most of their support came from fellow peers. Help-seeking from classmates and senior students has been linked to higher levels of self-regulated learning [13], while friendships may also influence exam performance, likely through informal sharing of study strategies [41]. However, this dynamic can also result in unequal access to valuable resources, as demonstrated in the subtheme of “peer support,” where senior students were reported to provide study skills guidance exclusively within their own societies. Similar patterns have been documented regarding the selective sharing of assessment materials, indicating that this issue extends beyond study skills alone [42]. Evidence from clinical skills education highlights that peer learning fosters a safe learning environment and aids SRL [43], whilst guidance from senior students has been identified as a positive predictor of SRL [13] . Given that future doctors must collaborate effectively, cultivating a culture of inclusivity and cooperation around study practices is crucial, and medical schools are well positioned to facilitate this.

The four most popular topics identified in the questionnaire, for which students sought study skills support, were those most specific to medicine, primarily focusing on exam preparation and content management. This finding aligns with the theme “specific study skills for medical school” and underscores the distinctive nature of medical education compared to other disciplines, highlighting that students prefer support tailored to the unique demands of their degree rather than generic university-wide resources. This preference may partly reflect the distinctive assessment methods in medicine, which extend beyond cognitive knowledge to include behavioral competencies, necessitating learning methods that may not have been part of students’ earlier educational experiences [44]. Furthermore, the vast volume of content in medical training—highlighted by the theme “medical school has a lot of content”—can be overwhelming and necessitates specific strategies for effective organization and retention.

Findings from both the questionnaire and focus group themes revealed that students highly value having access to a variety of study methods, including many which were already available at UCLMS or wider UCL. At the outset of the project, students in the research team recommended the creation of a centralized study skills page for the medical school on Moodle [22], the online platform used across UCL to deliver course material, and the data corroborated this suggestion from the wider student body. The research team, therefore, developed the page to provide UCLMS medical students with early and easy-to-access study skills support, directly addressing the need for improved accessibility highlighted in the theme “accessing support.” The page signposts and advertises existing resources, including links to book onto one-to-one or group “study skills clinics” with staff. In addition, informed by study findings, new resources were made by the students in the partnership such as anonymous group forums for peer-to-peer interaction, evidence-based articles on effective study techniques, and videos with study skills tips from other students. Overall, the platform provides diverse support, allowing students to access guidance that meets their individual learning needs.

### Strengths

This project offers several strengths. It adds to the existing literature on study skills support in medical education, with the advantage of a distinct emphasis on student perspectives. This was facilitated through multiple strategies, including the involvement of UCLMS students as members of the research team and as facilitators of the focus groups, which may have encouraged participants to speak more openly and candidly about the current provision of study skills support. Furthermore, while including the student authors’ names in recruitment emails may have introduced some response bias, it likely also enhanced engagement by motivating students with personal connections to the authors to participate.

Staff involvement in the project enabled a well-rounded, collaborative approach to understanding and addressing the study skills needs of UCLMS students, increasing the likelihood of successful implementation of proposed changes. For students, this collaboration with staff was particularly beneficial as it fostered a sense of being heard and valued. It allowed them to communicate their needs more effectively and see their suggestions translated into action, which, in turn, enhanced their engagement and motivation. Ultimately, this student–staff partnership established a mutually inclusive and supportive environment that strengthened communication and promoted a more meaningful dialogue around study skills support.

The development of the online resource itself also has several key strengths. It provides a centralized, easily accessible platform that supports a diverse student cohort by consolidating a wide range of study skills resources in one place. While primarily designed for students, the resource also offers UCLMS staff valuable insights into student needs and perspectives, meaning that in the future, changes can be made on a wider scale if necessary. Its interactive design and capacity for continuous updates also ensure the resource remains responsive and relevant to evolving student requirements, preventing it from becoming outdated over time.

### Weaknesses

The questionnaire had a low response rate, with just 6.9% (116/1682) of the medical student population participating, and 13.8% (16/116) of those respondents not completing all questions. This limits the generalizability of the findings and suggests results may disproportionately reflect students with a particular interest in study skills or those personally connected to the student researchers, whose names were included in the initial recruitment email. In addition, focus group recruitment relied on participants completing the questionnaire and then registering their interest via email at its conclusion, meaning the questionnaire dropout rate likely also contributed to the low focus group turnout. Finally, due to the small focus group sample, data saturation was not achieved, as novel themes emerged that could not be fully explored.

### Future Directions

Future study proposals would like to evaluate student and staff opinion on the study skills resources created and implemented. Overall, this study provides insight into the medical student perspective on study skills at UCLMS, identifying key areas of improvement and methods to target this. Similar initiatives could be effectively undertaken at other medical schools.

## Supplementary material

10.2196/65053 Multimedia Appendix 1 Table of contents outlining the questions within the questionnaire and answer options.

10.2196/65053 Multimedia Appendix 2 Table of contents of focus group questions and probing questions.

10.2196/65053 Multimedia Appendix 3 Table of contents of the results of the questions within the questionnaire. Given in mean, median, and percentage.

## Abbreviations

RUMS: Royal Free, University College and Middlesex Medical Students’ Association
SRL: self-regulated learning
UCL: University College London
UCLMS: University College London Medical School
