# Supplementary material for: Assessing and Improving Study Skills Support in Medical Education Through a Student-Staff Partnership: Mixed Methods Approach
Source: JMIR Med Educ. 2025 Sep 3;11:e65053. doi: 10.2196/65053 (PMC12408056; doi:10.2196/65053)
Supplement: Multimedia Appendix 1 [file mededu-v11-e65053-s001.pdf]

Supplementary Table 1 – Questions within the Questionnaire

Question Number	Question	Responses
1	**What is your current year of study? Options:**	Year 1<br>Year 2<br>Year 4<br>Year 5<br>Year 6
2	**How confident do you currently feel in the following study skills? Responses in Likert Scale (1 to 5). Options include:**	Time management<br>Organisation<br>Exam preparation and technique<br>Obtaining reliable sources of information<br>Retaining information<br>Study skills specific to medical degree (e.g. practical exams, navigating clinical years)<br>Study skills and mental health (e.g. work-life balance, feeling overwhelmed)
3	**Which resources do you use to support your study skills? Options include:**	Synchronous or asynchronous lectures from UCLMS<br>Self-directed learning activities from other UCL resources<br>One to one support: from staff or peers<br>One to one support: from peers<br>Study skills clinic<br>Personal tutor<br>Transition mentor<br>None of the above
4	**Which of the following study skills have you received support for whilst at UCLMS? Options include:**	Time management<br>Organisation<br>Exam preparation and technique<br>Obtaining reliable sources of information<br>Retaining information<br>Study skills specific to medical degree (e.g. practical exams, navigating clinical years)<br>Study skills and mental health (e.g. work-life balance, feeling overwhelmed)<br>None of the above
5	**Have you accessed the central UCL study skills page? If so, please rate its effectiveness. Total out of 100.**	Part 1 of question: Yes<br><br>If yes to part one, move onto part 2: Likert Scale (1 to 5) for effectiveness
6	**How often have you received study skills support at whilst at UCLMS? Options include:**	Never<br><1 a year<br>1-2 times a year<br>3-5 times a year<br>>5 times a year

7	**Please Rate the effectiveness of how study skills support was delivered: Likert Scale (1 to 5). Options include:**
	Synchronous or asynchronous lectures from UCLMS<br>Self-directed learning activities from other UCL resources<br>One to one support: from staff<br>One to one support: from peers<br>Study skills clinic<br>Personal tutor<br>Transition mentor
8	**How often would you like to have study skills support delivered? Options include:**
	Never<br>Once in the MBBS programme<br>Once in pre-clinical, once in clinical years<br>Yearly<br>Every term<br>Others, please specify
9	**How would you like to have study skills support delivered? Options include:**
	Synchronous or asynchronous lectures from UCLMS<br>Self-directed learning activities from other UCL resources<br>One to one support: from staff<br>One to one support: from peers<br>Peer-peer group teaching<br>Small group tutoring<br>Study skills clinic<br>Personal tutor<br>Transitional Mentor
10	**What topics would you to be included in the above study skills support? Options include**
	Time management<br>Organisation<br>Exam preparation and technique<br>Obtaining reliable sources of information<br>Taking in new information<br>Retaining information<br>Study skills specific to medical degree (e.g. practical exams, navigating clinical years)<br>Study skills and mental health (e.g. work-life balance, feeling overwhelmed)<br>None of the above
11	**How useful would a central Moodle page for study skills resources be for you? Likert Scale (1 to 5)**
12	**Please let us know of any comments or suggestions regarding study skill support at UCLMS: Free text answer**
