# Supplementary material for: Assessing and Improving Study Skills Support in Medical Education Through a Student-Staff Partnership: Mixed Methods Approach
Source: JMIR Med Educ. 2025 Sep 3;11:e65053. doi: 10.2196/65053 (PMC12408056; doi:10.2196/65053)
Supplement: Multimedia Appendix 2 [file mededu-v11-e65053-s002.pdf]

Supplementary Table 2 – Focus group questions

Question Number	Question	Responses
1	**What do you understand by the term study skills?**	
		If needed, clarify definition – for the purposes of this focus group, study skills will include time management, note-taking, which resources to use, exam technique etc.
2	**Can you describe your experience of study skills support or teaching at UCLMS?**	
3	**From the questionnaire, it seems that students would like small group teaching, lectures (asynchronous/synchronous) and 1:1 support from staff.**	
		What are your thoughts on this?<br>Explore each route of delivery
4	**From the questionnaire, the most requested content of study skills support were exam preparation and study skills specific to medical degree.**	
		What are your thoughts on this?
5	**What do you think about the creation of a Central Moodle page for Study Skills resources?**	
		What do you think it should include?
6	**What do you know about UCLMS's Study Skills Clinics?**	
		How did you hear about the study skills clinic?<br>Is this something you would consider attending?<br>What would be useful in the Study Skills Clinic for you?
7	**Would you find one to one mentoring (peer or staff) useful?**	
8	**Is there anything that we haven't mentioned that you think would be really important to?**	
		Limit if needed
9	**Probing questions to help facilitate discussion if required:**	
		Could you tell me more about that?<br>Could you expand on that?<br>Can you give any specific examples?<br>How so?<br>In what way?
