# Supplementary material for: Assessing and Improving Study Skills Support in Medical Education Through a Student-Staff Partnership: Mixed Methods Approach
Source: JMIR Med Educ. 2025 Sep 3;11:e65053. doi: 10.2196/65053 (PMC12408056; doi:10.2196/65053)
Supplement: Multimedia Appendix 3 [file mededu-v11-e65053-s003.pdf]

Supplementary Table 3 – Demographical and Questionnaire Results

Question Number	Question	Responses
1	**What is your current year of study? Total: 116**	
	1	22
	2	32
	4	20
	5	17
	6	25
2	**How confident do you currently feel in the following study skills? Likert Scale (1 to 5): Median (mean)**	
	Time management	3.0 (3.34)
	Organisation	4.0 (3.56)
	Exam preparation and technique	3.0 (2.94)
	Obtaining reliable sources of information	4.0 (3.63)
	Retaining information	3.0 (2.93)
	Study skills specific to medical degree (e.g. practical exams, navigating clinical years)	3.0 (2.93)
	Study skills and mental health (e.g. work-life balance, feeling overwhelmed)	3.0 (2.97)
3	**Which resources do you use to support your study skills? Total: 116. Results given in number (percentage, %)**	
	Synchronous or asynchronous lectures from UCLMS	86 (74.1)
	Self-directed learning activities from other UCL resources	67 (57.8)
	One to one support: from staff or peers	14 (12.1)
	One to one support: from peers	55 (47.4)
	Study skills clinic	5 (4.3)
	Personal tutor	17 (14.7)
	Transition mentor	9 (7.8)
	None of the above	3 (2.6)
4	**Which of the following study skills have you received support for whilst at UCLMS? Total: 116. Results given in: number (percentage, %)**	
	Time management	15 (12.9)
	Organisation	15 (12.9)
	Exam preparation and technique	46 (39.7)
	Obtaining reliable sources of information	41 (35.3)
	Retaining information	11 (9.5)
	Study skills specific to medical degree (e.g. practical exams, navigating clinical years)	53 (45.7)
	Study skills and mental health (e.g. work-life balance, feeling overwhelmed)	36 (31.0)
	None of the above	36 (31.0)
5	**Have you accessed the central UCL study skills page? If so, please rate its effectiveness. Total out of 100.**	
	Part 1 of question: Yes	20 (out of 100)
	If yes to part one, move onto part 2: Likert Scale (1 to 5) for effectiveness; Median (mean)	3.0 (3.3)

**6**	**How often have you received study skills support at whilst at UCLMS? Total: 100. Results given in: number (percentage, %)**	
	Never	68
	<1 a year	7
	1-2 times a year	19
	3-5 times a year	5
	>5 times a year	2
**7**	**Please Rate the effectiveness of how study skills support was delivered: Likert Scale (1 to 5): Median (mean) result**	
	Synchronous or asynchronous lectures from UCLMS	3.0 (2.67)
	Self-directed learning activities from other UCL resources	3.0 (2.76)
	One to one support: from staff	3.0 (2.67)
	One to one support: from peers	3.14 (3.34)
	Study skills clinic	2.0 (2.2)
	Personal tutor	3.0 (2.50)
	Transition mentor	2.83 (2.24)
**8**	**How often would you like to have study skills support delivered?**	
	Never	3
	Once in the MBBS programme	5
	Once in pre-clinical, once in clinical years	16
	Yearly	34
	Every term	40
	Others, please specify	
**9**	**How would you like to have study skills support delivered? Total: 100, results in percentage (%)**	
	Synchronous or asynchronous lectures from UCLMS	43
	Self-directed learning activities from other UCL resources	24
	One to one support: from staff	41
	One to one support: from peers	18
	Peer-peer group teaching	34
	Small group tutoring	56
	Study skills clinic	33
	Personal tutor	24
	Transitional Mentor	13
**10**	**What topics would you to be included in the above study skills support? Total: 100, Results given in: number (percentage, %)**	
	Time management	44
	Organisation	42
	Exam preparation and technique	83
	Obtaining reliable sources of information	39
	Taking in new information	58
	Retaining information	69
	Study skills specific to medical degree (e.g. practical exams, navigating clinical years)	72
	Study skills and mental health (e.g. work-life balance, feeling overwhelmed)	50
	None of the above	nil
**11**	**How useful would a central Moodle page for study skills resources be for you? Likert Scale (1 to 5): Median (mean)**	
		4.0 (3.66)
